# Remarks on the Pathology and Treatment of the Pneumonia of Early Infancy

**Published:** 1883-03

**Authors:** 


### REMARKS ON THE PATHOLOGY AND TREATMENT OF THE PNEUMONIA OF EARLY INFANCY.

I desire to submit for your consideration to-day some remarks on the pathology and treatment of pneumonia as it occurs in the first six months of infancy. All medical men are fully aware of the great mortality attending this affection in early life, and thoroughly appreciate the difficulties in the way of its successful treatment. Statisticians inform us that one-third of all infants born alive perish before they reach the fifth year of age. This is an enormous rate of mortality, and pneumonia is certainly responsible for a very considerable proportion of it. Indeed, in point of mortality it will rank with most of the grave diseases of infancy, and in an equal degree deserves our serious consideration.

There is an impression abroad that medical men do not comprehend clearly and scientifically the diseases of infancy and their treatment, but depend very much upon mere personal observation and experience, and trust to good fortune for results. It may be that we do not attach due importance to these apparently trivial matters, not reflecting on the vast influence which these questions must exert on the future progress of our race. It is from this embryo material which we visit and prescribe for in our daily rounds of professional duty that springs the splendid man, with all his varied attributes of intellect, whether it takes the direction of oratory, science, poetry, art or military heroism, and lovely woman, but little less beautiful in character and person than the angels, with all of her purer and better instincts exerting that refining and elevating influence on our moral natures which no other earthly power but woman can.

One of the missions of our profession, and a duty imperative on us, is to do all in our power to lessen this fearful rate of infantile mortality. One of the great needs of our Southern country is a population sufficient in numbers, in health and vigor to develop our magnificent resources, and build up our important interests. If we now had that superb element of population which was sacrificed in the late war, we would need nothing further to restore us to prosperity.

But it is a most gratifying reflection to know that our knowledge of the pathology and treatment of infantile diseases is constantly and steadily advancing towards a plain of more thorough scientific accuracy. I can observe marked changes in these respects for the better in the past twenty years. Yet, this department of our profession is still far from being complete.

*Forms of pneumonia common in infancy.*—There are two forms of pneumonia to which very young infants are more particularly subject—the lobar and catarrhal. While *lobar* pneumonia in the infant does not materially differ from that in the adult, in my experience, the attending râle is rather coarser, and more on the sub-crepitant order, and the dullness on percussion is not as complete. *Catarrhal* pneumonia—formerly existing under the names of suffocative catarrh and capillary bronchitis—is the most common to which infancy is liable, and the most dangerous to life, as it usually involves both entire lungs. In such cases the secretion of mucus is so rapid, and its accumulation so extensive as to cut off very speedily all ingress of atmospheric air into the air cells. Hence, at a very early stage of these cases we observe the manifestations of carbonic acid poisoning, or cyanosis, and a disposition to drowsiness. I have seen cases threatened with suffocation in twelve hours after the onset of the disease.

In proportion to the accumulation of carbonic acid in the blood, the disposition to narcosis becomes profound, so that the infant appears as if suffering from a poisonous dose of opium. I saw, on one occasion, a little infant of only a week old with catarrhal pneumonia, which presented a complexion almost like indigo. The tendency of carbonic acid poisoning is not only to cause sleep, but also to suppress cough.

*The character and rate of respiration.*—Probably in no other disease does the respiration rate reach so high a standard as in the pneumonia of early infancy. I am very sure that I have seen in an infant a few days old, with pneumonia, the frequency of respiration reach one hundred per minute. In proportion as the air cells and bronchioles fill with accumulating exudation, the inspirations become shorter in duration and interval, and, of course, the respiratory acts are more frequent and less efficient. In these very grave cases of infantile pneumonia, the acts of respiration become so rapid and short that the inspired air, to the observer, does not appear to penetrate more than one-third of the lungs. We often hear adult patients with serious pectoral affections use the expression that they could inhale the air to a certain point in the chest only, denoting precisely the line of demarkation between the sound and diseased tissue. This is literally true of all chest affections causing obstruction. No sooner does the inspired air enter the cells than a decided sense of relief follows, and the chest walls expand in corresponding degree. If we expose the chest of an infant suffering from extensive catarrhal pneumonia, it will at once be observed that the walls around the bases of the lungs are not expanding as they should do, but that the muscles of the apex of the chest, also those of the neck and back, are thrown into an extraordinary state of action by the powers of volition, for the purpose of still further dilating the lungs.

*The character of cough in infantile pneumonia.*—In proportion to the extent and gravity of pulmonic disease, the attendant cough will diminish in frequency and force, and decline in aiding expectoration. A very serious danger to be apprehended during the progress of intantile pneumonia is the partial or entire suppression of cough. This may be brought about either by the effects of carbonic acid poisoning, or narcosis from the too free use of opium. Under these circumstances, the infant rarely voluntarily takes those deep inspirations which distend the lungs, aërate the blood, and excite cough and expectoration.

In this connection, I think it proper to state that in those cases where the cough is either suppressed or insufficient in promoting expectoration, it is my invariable custom to resort to artificial means for the purpose of stimulating that function. The agents used are decidedly antiseptic in character, and exert the double influence of disinfecting the accumulated matter in the bronchial tubes, and exciting these tubes to active contraction, and the expulsion of the offending cause, to the great relief of the little patient. My habit invariably, in this class of cases, is to use every three or four hours, or even oftener if necessary, by means of a continuous spray-producer, a solution composed of

℞. Alcohol..........................℥j

Water.............................℥ij

Carbolic acid......................ʒss

Bicarbonate soda..................ʒj

Salicylic acid.....................ʒj

Chloral hydrate...................℈ss. M.

The atmosphere surrounding the head and chest of the patient is to be charged with the spray of this solution as often as it may be necessary to excite cough and expectoration for a few minutes at each time.

The invariable result in my experience has been the restoration of cough and free expectoration, for the time being, with improvement in the breathing and complexion. By the persevering use of this device, I feel sure that on several occasions I saved the lives of children that would otherwise have been lost.

I remember, on a certain occasion, having for a patient a most interesting little boy of two years old, who had endeared himself to all by his exceeding brightness of character and amiability of temper. He was suffering from a fearful attack of double catarrhal pneumonia and wooping-cough combined. He suffered terribly for many days from suppression of expectoration and great oppression of breathing. The cough was very slight, and only occasional. There were crepitant râles over the entire of both lungs. The pulse and rate of respiration were exceedingly frequent, and the complexion of a dusky hue. For the purpose of restoring cough and expectorating, the atomiser was used freely every three hours, and at times oftener—not only with infinite temporary relief, but, I am convinced, with the ultimate result of saving the life of the patient.

To stand by the bedside of these helpless little creatures, suffering a fearful degree of agony from oppression of breathing, with a respiration of seventy per minute, and a pulse amounting to one hundred and sixty or seventy, and witness the relief afforded by the simple use of the antiseptic spray, is exceedingly gratifying.

*Character and Rate of Pulse.*—Dr. West says that it is unusual for the pulse in infantile pneumonia to reach one hundred and eighty per minute. Other authors, with the more accurate methods of calculating at present, say that it frequently attains a rate of two hundred, and even two hundred and twenty-five in bad cases. The rate of the pulse and respiration never reaches these high figures in the adult. Hence, we have a state of affairs, so far as these questions are concerned, differing entirely from the pneumonia of the adult.

Now, the question is, what are the effects of these very high rates of the pulse and respiration on the progress of the case? During the very rapid action of the small and feeble infantile heart in pneumonia, each diastole is so brief and imperfect that very few drops of venous blood can be received into the right chambers of the heart at each pulsation. Thus, while the heart may be acting at the rate of two hundred per minute, each two pulsations are not equal in efficiency in propelling the column of blood forward through the circle, to one pulsation when the organ is acting at the rate of one hundred per minute. The practical result is the blood constantly tends to accumulate in the venous system, producing cyanosis, and its consequences, carbonic acid poisoning and narcosis, with labored breathing; while the arterial system, failing to receive its due share of oxygenated blood, induces the small, feeble and frequent pulse characteristic of this condition, with the extreme general prostration which always attends this state. The pulse in infantile disease not uncommonly attains a rate of frequency more than double that of health, and yet this may be followed by a complete restoration.

Medical men, from practical experience, well know how delicate the infantile system is, and yet how wonderfully elastic it is in its recuperative energies. The nervous system of children, whether volitional or sensitive, are exceedingly excitable. But the ganglionic system is intensely emotional, giving rise to that remarkable excitability of the heart which we see manifested in our daily intercourse with these little patients, elevating the pulse, even in slight febrile conditions, to a fearful degree of frequency, the violent impulse of the heart resembling that from hypertrophy, while every superficial artery in the body can be seen in violent commotion. This remarkable excitability of the varied component parts of the nervous system of infants and children, with their emotional tendencies, constitutes one of the most important considerations in the history and treatment of infantile diseases. These peculiar characteristics are prone to mask all other symptoms, whether of functional or organic disease, to such an extent as to obscure the real difficulty and mislead us in forming our opinions. The extent of pneumonic inflammation which would elevate the rate of respiration to thirty and the pulse to one hundred and twenty in the adult, would, in the young infant, send the rate of respiration up to a hundred, and the pulse to the neighborhood of two hundred per minute. Taking the adult rates in these respects as our standard for guidance, the excessive rates above these in the infant must be regarded as attributable to the remarkable excitability of the sympathetic system of nerves peculiar to that period of life.

A knowledge of this fact in the treatment of infantile disease is of infinite importance. If we were to use violent or depletive means for these explosions of nervous force and excitability in the inflammatory diseases of infancy, we would simply induce a state of general prostration without removing the cause; whereas, if means are used to control this extraordinary erethism of the sympathetic system, and through these reducing the excessive cardiac and respiratory action, we at once bring the state of the system to a safe point for the resolution of the local disease.

The tendency to clonic convulsions in the febrile affections of infants is another example of explosion of nervous force and excitability in the volitional and reflex centres, as the great frequency of the action of the heart and respiration represents that of the ganglionic system.

*Treatment.*—With all our preconceived ideas of the prompt and vigorous measures necessary to subdue a violent attack of pneumonia in the adult, I confess to have formerly approached the treatment of this dangerous disease in the feeble and delicate infant, whose life apparently hangs upon a mere thread, with a considerable degree of misgiving. Since adopting the method which I now resort to in these cases, my practice has been far more satisfactory and successful than formerly.

The great and pressing objects to be accomplished for the speedy relief of the patient in these cases, are to disgorge the bronchioles of superabundant mucus, reduce frequency of the pulse and respiration, and at the same time sustain the strength of the heart and its force of action. Nothing should be done in this affection to impair the force of the heart's action. Fortunately for us, there are remedies at hand which will not only reduce abnormal frequency of the pulse and respiration, but will, at the same time, give tone and vigor to every pulsation of the heart. With these objects accomplished, and proper sustenance of the patient, resolution of the pulmonary engorgement and inflammation will usually follow.

I cannot present a better illustration of the line of practice which I have pursued in these cases in recent years than by giving in detail the history of a case of double pneumonia, very alarming in character, occurring in a little infant only three weeks old:

When I first saw this case, the pulse was so rapid and feeble that I could not even approximate the rate, and scarcely detect it at the wrist. The respiration must have been nearly one hundred per minute. The complexion was completely cyanosed. There were universal subcrepitant râles throughout both lungs, and dulness over both bases. The chest walls, on inspection, scarcely expanded at all. No one supposed that the little creature could survive but a few hours. The family discussed the question seriously whether or not, the chances of life being so limited, it was justifiable to vex and annoy the infant with treatment. At my earnest solicitation, vigorous measures were put in practice, and the result was the saved life of a boy now several years old, and in the full vigor of health. A warm mustard bath was ordered to be used every four hours, to be followed by brisk frictions with a dry flannel. The patient was to take a teaspoonful of the following mixture every three hours, viz.:

℞. Liq. ammon. acetatis............ʒii.

Tinct. belladonna...............gtt. xii

Tinct. digitalis..................gtt. xii

Spts. ammon. arom..............ʒiss

Vin. ipecac.......................gtt. xvi

Aquæ..........................ʒx

Syr. acaciæ.....................ʒvi. M.

The antiseptic spray was also ordered every four hours. A few drops of good brandy were given with each dose of the mixture. Small dry cups were applied over the dorsal portion of the chest. The effects of this treatment in forty-eight hours were marvellous.

Of all those agents for slowing the excitable and feeble infantile heart in pneumonia, and reducing the high pulse rate down to the natural standard, and imparting vigor and tone to its action, digitalis stands first on the list. It has not the rapid and dangerous action of veratrum and aconite, but in the end is just as efficient.

I regard belladonna, used in connection with digitalis, as a most important element in the therapeusis of infantile pneumonia. In this connection it exerts a most soothing and quieting influence over the irritable condition of the sympathetic system, not only aiding the digitalis in composing the action of the heart, but especially reducing the high rate of respiration, and thereby affording relief to the oppression of breathing. Belladonna is well known to exert a remarkable influence in curtailing superabundant secretion, whether from the skin, intestines or lungs.

I feel sure that this power is exerted in a very decided degree in gradually diminishing the excessive secretion of mucus in catarrhal dneumonia, and thereby preventing renewed pulmonary obstruction. It is one of the few agents in our possession which accomplishes this object without either suppressing cough or embarrassing the respiration, or aggravating the bronchial inflammation.

The mild preparations of ammonia are particularly valuable in liquifying viscid mucus and hastening its expulsion. As a sustainer of the exhausted and flagging nervous energies of the infantile constitution, it is almost invaluable in the treatment of this disease.

The wine of ipecac, in my judgment, should never be dispensed with in the treatment of the pneumonia of infancy. Whenever given in minute doses continuously, it acts as a potent promoter of expectoration, inducing contraction of the bronchial tubes, and the gradual expulsion of mucus.

In a most obstinate case of catarrhal pneumonia, in an aged woman, which resisted every known remedy, with constantly increasing severity of symptoms, accompanied by a very irritable stomach, in utter despair I determined to put the case on minute doses of ipecac wine —say two drops every three hours. In twenty-four hours there was marked improvement in the respiration and pectoral oppression, and in the freedom and ease of expectoration, with relaxation of skin. Under this remedy, slightly increased in dose, the patient entirely recovered.

We know now that ipecac exerts a very extended influence over the entire sympathetic and vaso-motor systems, and by quieting nervous excitement and vascular action, it controls hæmorrhage and regulates secretion. The combination of the alkalies—ammonia, soda or potash —with the ipecac, particularly the former, constitutes, in my experience, the very best of means for enabling the bronchial tubes to expel their contents in pectoral inflammations. Opium, when the moist râles are abundant in infantile pneumonia or bronchitis, is inadvisable and dangerous.

In forming an opinion of the admissibility of opium in these cases, we must be guided by the extent of tissue involved and the extent of râles present. If these are extensive, then opium must be discarded. The bromides here are specially applicable for quieting nervous restlessness and procuring sleep.

In lobar pneumonia, when the muco-febrinous secretion is very tenacious, I have used the iodide of potash, iodide of ammonia, spirits of ammonia and ipecac with the best effect.

Dr. J. E. Chancellor reported a case in his practice which confirms Dr. Brown's views.

